# CCT6A alleviates pulmonary fibrosis by inhibiting HIF-1α-mediated lactate production

**DOI:** 10.1093/jmcb/mjae021

**Published:** 2024-05-17

**Authors:** Peishuo Yan, Kun Yang, Mengwei Xu, Miaomiao Zhu, Yudi Duan, Wenwen Li, Lulu Liu, Chenxi Liang, Zhongzheng Li, Xin Pan, Lan Wang, Guoying Yu

**Affiliations:** State Key Laboratory of Cell Differentiation and Regulation, Henan International Joint Laboratory of Pulmonary Fibrosis, Henan Center for Outstanding Overseas Scientists of Organ Fibrosis, Pingyuan Laboratory, College of Life Science, Henan Normal University, Xinxiang 453007, China; State Key Laboratory of Cell Differentiation and Regulation, Henan International Joint Laboratory of Pulmonary Fibrosis, Henan Center for Outstanding Overseas Scientists of Organ Fibrosis, Pingyuan Laboratory, College of Life Science, Henan Normal University, Xinxiang 453007, China; State Key Laboratory of Cell Differentiation and Regulation, Henan International Joint Laboratory of Pulmonary Fibrosis, Henan Center for Outstanding Overseas Scientists of Organ Fibrosis, Pingyuan Laboratory, College of Life Science, Henan Normal University, Xinxiang 453007, China; State Key Laboratory of Cell Differentiation and Regulation, Henan International Joint Laboratory of Pulmonary Fibrosis, Henan Center for Outstanding Overseas Scientists of Organ Fibrosis, Pingyuan Laboratory, College of Life Science, Henan Normal University, Xinxiang 453007, China; State Key Laboratory of Cell Differentiation and Regulation, Henan International Joint Laboratory of Pulmonary Fibrosis, Henan Center for Outstanding Overseas Scientists of Organ Fibrosis, Pingyuan Laboratory, College of Life Science, Henan Normal University, Xinxiang 453007, China; State Key Laboratory of Cell Differentiation and Regulation, Henan International Joint Laboratory of Pulmonary Fibrosis, Henan Center for Outstanding Overseas Scientists of Organ Fibrosis, Pingyuan Laboratory, College of Life Science, Henan Normal University, Xinxiang 453007, China; State Key Laboratory of Cell Differentiation and Regulation, Henan International Joint Laboratory of Pulmonary Fibrosis, Henan Center for Outstanding Overseas Scientists of Organ Fibrosis, Pingyuan Laboratory, College of Life Science, Henan Normal University, Xinxiang 453007, China; State Key Laboratory of Cell Differentiation and Regulation, Henan International Joint Laboratory of Pulmonary Fibrosis, Henan Center for Outstanding Overseas Scientists of Organ Fibrosis, Pingyuan Laboratory, College of Life Science, Henan Normal University, Xinxiang 453007, China; State Key Laboratory of Cell Differentiation and Regulation, Henan International Joint Laboratory of Pulmonary Fibrosis, Henan Center for Outstanding Overseas Scientists of Organ Fibrosis, Pingyuan Laboratory, College of Life Science, Henan Normal University, Xinxiang 453007, China; State Key Laboratory of Cell Differentiation and Regulation, Henan International Joint Laboratory of Pulmonary Fibrosis, Henan Center for Outstanding Overseas Scientists of Organ Fibrosis, Pingyuan Laboratory, College of Life Science, Henan Normal University, Xinxiang 453007, China; State Key Laboratory of Cell Differentiation and Regulation, Henan International Joint Laboratory of Pulmonary Fibrosis, Henan Center for Outstanding Overseas Scientists of Organ Fibrosis, Pingyuan Laboratory, College of Life Science, Henan Normal University, Xinxiang 453007, China; State Key Laboratory of Cell Differentiation and Regulation, Henan International Joint Laboratory of Pulmonary Fibrosis, Henan Center for Outstanding Overseas Scientists of Organ Fibrosis, Pingyuan Laboratory, College of Life Science, Henan Normal University, Xinxiang 453007, China

**Keywords:** IPF, CCT6A, lactate signaling, metabolism, HIF-1α

## Abstract

Idiopathic pulmonary fibrosis (IPF) is a lethal progressive fibrotic lung disease. The development of IPF involves different molecular and cellular processes, and recent studies indicate that lactate plays a significant role in promoting the progression of the disease. Nevertheless, the mechanism by which lactate metabolism is regulated and the downstream effects remain unclear. The molecular chaperone CCT6A performs multiple functions in a variety of biological processes. Our research has identified a potential association between CCT6A and serum lactate levels in IPF patients. Herein, we found that CCT6A was highly expressed in type 2 alveolar epithelial cells (AEC2s) of fibrotic lung tissues and correlated with disease severity. Lactate increases the accumulation of lipid droplets in epithelial cells. CCT6A inhibits lipid synthesis by blocking the production of lactate in AEC2s and alleviates bleomycin-induced pulmonary fibrosis in mice. In addition, our results revealed that CCT6A blocks HIF-1α-mediated lactate production by driving the VHL-dependent ubiquitination and degradation of HIF-1α and further inhibits lipid accumulation in fibrotic lungs. In conclusion, we propose that there is a pivotal regulatory role of CCT6A in lactate metabolism in pulmonary fibrosis, and strategies aimed at targeting these key molecules could represent potential therapeutic approaches for pulmonary fibrosis.

## Introduction

Idiopathic pulmonary fibrosis (IPF) has unknown etiology, unclear pathology, and high mortality. Its characteristics include excessive accumulation of extensive extracellular matrix (ECM) in alveoli, solid fibrosis of alveolar structure (Raghu et al., 1999; Richeldi et al., 2017). The disease is often diagnosed in people over 65 years old. The patients usually show dyspnea or dry cough. In the late stage of the disease, even the interruption of gas exchange will lead to death (Lederer and Martinez, 2018). Presently, it is generally thought that repeated epithelial cell damage and over repair are key to the pathogenesis of IPF. The abnormal activation of AEC2s, the recruitment of interstitial cells, and the secretion of various fibrotic growth factors and cytokines. Lots of fiber-promoting mediators lead to the recruitment of myofibroblasts from different sources in the alveoli, leading to excessive extracellular matrix deposition and changes in the structure and hardness of the alveoli, destroying the lung parenchyma (Noble et al., 2012; Martinez et al., 2017). Only 3–5 years median survival after IPF diagnosis (Kolb et al., 2018). Currently, there are only two drugs approved by the FDA for IPF. However, the drugs hardly completely block the progression of lung fibrosis. Therefore, clarifying its pathogenesis and being able to diagnose and treat it effectively in time is the biggest challenge facing clinical and scientific research fields.

One of the main characteristics of IPF patients is metabolic disorders in lung tissues, including the impairment of mitochondrial function and the increase in glycolysis (Bueno et al., 2020; Larson-Casey et al., 2020; Manevski et al., 2020). Pharmacological or genetic inhibition of PFKFB3 suppressed cellular glycolysis and blunted the differentiation of lung fibroblasts into myofibroblasts (Xie et al., 2015; Chen et al., 2021). Lactate, traditionally regarded as a metabolic waste product, is now recognized as a signaling molecule with autocrine, paracrine, and endocrine-like effects, referred to as ‘lactomone’ (Brooks, 2009, 2020). Researchers have found that lactate levels are abnormally increased in the lungs and serum of people with IPF, also lactate activates TGF-β1 in a pH-dependent manner *in vitro*, thereby inducing the differentiation of lung fibroblasts (Kottmann et al., 2012). Research shown that an abnormal upregulation of the lactate level in bronchoalveolar lavage fluid (BALF) of bleomycin (BLM)-treated mice. p300 could mediate lactate-induced histone lactylation in macrophages *in vitro*, thereby increasing the expression of fibrosis-promoting genes (Cui et al., 2021). Although the crucial regulation of lactate in the development of IPF is unquestionable, the source of lactate and its possible additional metabolic regulation are still unknown.

Molecular chaperones are a class of intracellular molecules with rich functions. At present, there is no relevant report on pulmonary fibrosis. In our previous study, we found that CCT6A, a member of the chaperonin-containing TCP1 complex (CCT), regulated lactate metabolism in lung fibroblasts (Wang et al., 2023). Considering that the metabolic activity of AEC2 is more exuberant, it may be one of the main sources of lactate in the lung. We speculate that CCT6A has a more critical role in epithelial cells. Our research clarified the mechanism by which lactate promotes pulmonary fibrosis, and CCT6A significantly inhibited the progression of pulmonary fibrosis by reducing the excessive production of lactate in AEC2s. In general, our data demonstrate the importance of CCT6A in maintaining normal metabolism of AEC2s and provide a direction for potential therapeutic interventions to treat IPF.

## Results

### Increased CCT6A expression in AEC2s of fibrotic lung

**Figure 1:**
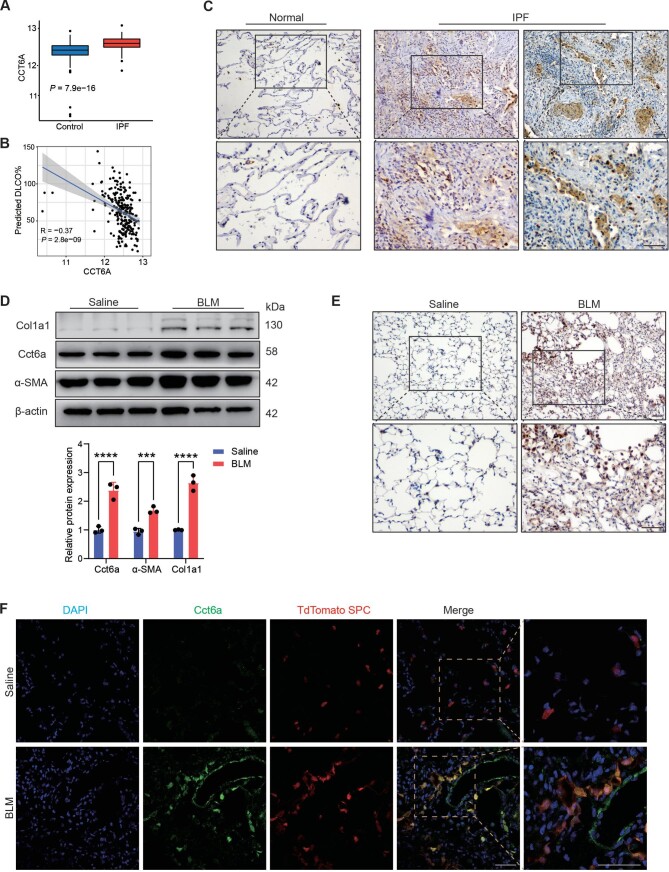
CCT6A is highly expressed in lung tissues of IPF and BLM-induced lung fibrosis in mice. (**A**) Publicly available array data (GSE47460) showing CCT6A expression levels in lung tissues of IPF (*n* = 160) and control subjects (*n* = 108). (**B**) Correlation between CCT6A expression level and the diffusion capacity for carbon monoxide, determined by Pearson Correlation. (**C**) Representative images of immunohistochemical analysis of CCT6A level in human lung tissue samples (*n* = 4). Boxed regions are magnified from the panel. Scale bar, 50 μm. (**D**) Immunoblot analysis of Cct6a, α-SMA, and Col1a1 levels in lung tissues of BLM-induced lung fibrosis (*n* = 3) and control group (*n* = 3). (**E**) Representative images of immunohistochemistry analysis of Cct6a level in mouse lung tissue samples (*n* = 4). Boxed regions are magnified from the panel. Scale bar, 50 μm. (**F**) Representative images of co-immunostaining of Cct6a and SPC (an AT2 marker) in mouse lung tissue samples (*n* = 5). Scale bar, 50 μm. ***P < 0.001, ****P <0.0001.

Our previous study demonstrated that CCT6A was significantly increased in serum derived from IPF patients compared with control subjects. Re-analysis of the publicly available dataset (GSE47460; Vukmirovic et al., 2017) revealed significant upregulation of CCT6A mRNA in the lungs of IPF (Figure 1A). Then, we assessed and found a significant negative correlation between CCT6A gene expression and disease severity, as reflected by the diffusion capacity for carbon monoxide (DLCO% predicted, Figure 1B). An increased CCT6A immunohistochemical staining was observed in the lung tissue from patients with IPF compared with healthy control subjects, and CCT6A was mainly expressed in the alveolar epithelium surrounding the fibroblastic foci in IPF lung tissue but not in the normal alveolar epithelium (Figure 1C). To further address the role of CCT6A in pulmonary fibrosis, we detected the expression of CCT6A in the lungs of mice with experimental pulmonary fibrosis induced by BLM. Similarly, the level of Cct6a in the lungs of mice treated with BLM for 21 days was significantly increased, and we also noted markedly higher expression levels of Col1a1 and α-SMA, markers of fibrosis, in the BLM group (Figure 1D). We further examined lung sections from mice after BLM injection. Consistent with the results in human lung tissues, compared with the control group, the CCT6A in lung tissue of mice induced by BLM was significantly increased (Figure 1E). Co-immunostaining showed increased positive staining of CCT6A located in the epithelial area in fibrotic lung tissues, manifesting as high levels of CCT6A revealed by containing CCT6A with SPC (Figure 1F).

### CCT6A suppresses lactate production and reverses the lipid accumulation induced by lactate in AEC2s

**Figure 2:**
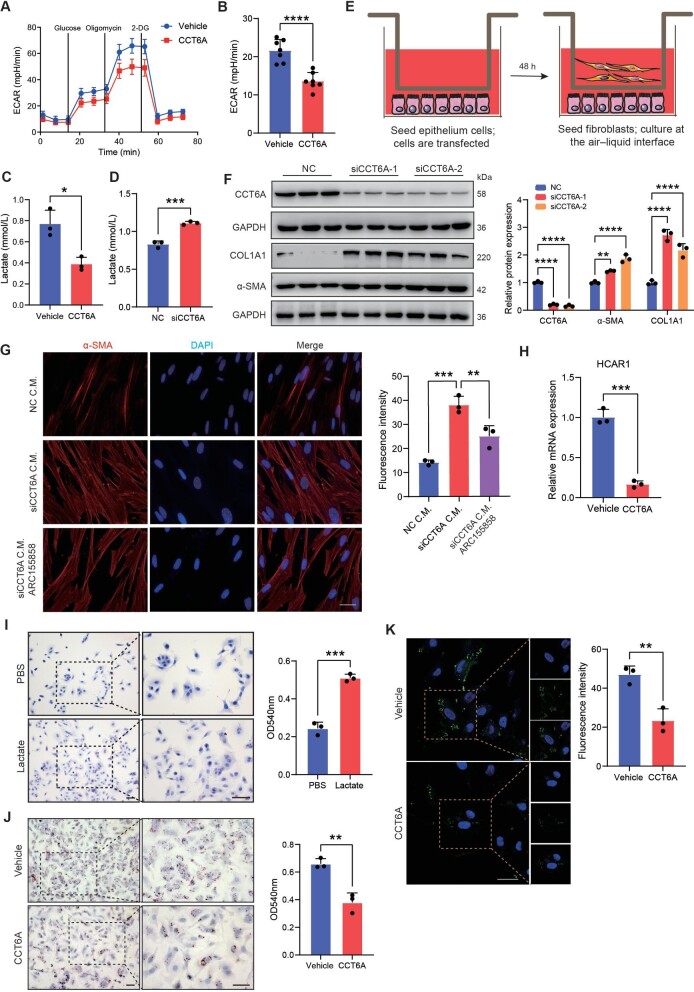
CCT6A suppresses lactate production and reverses the lipid accumulation in alveolar epithelial cells. (**A** and **B**) Real-time ECAR (mpH/min) analysis of cells transfected with CCT6A plasmid under basal conditions followed by addition of glucose (10 mM), oligomycin (1 μM), and 2-DG (50 mM) as indicated. (**C** and **D**) Extracellular lactate production in AEC2s transfected with CCT6A plasmid or CCT6A plasmid. (**E**) Schematic of a co-culture system of transfected epithelial cells and fibroblasts. (**F**) Western blotting and quantification of α-SMA and COL1A1 protein expression in fibroblasts co-cultured with siCCT6A-transfected epithelial cells. (**G**) Immunofluorescence staining and quantification of α-SMA in fibroblasts co-cultured with siCCT6A-transfected epithelial cells in the presence or absence of ARC155858. Scale bar, 50 μm. (**H**) qPCR analysis of HCAR1 mRNA expression levels in transfected epithelial cells. (**I** and **J**) Oil Red O staining and quantification of epithelial cells treated with 20 mM lactate or transfected epithelial cells following lactate induction. Boxed regions are magnified from the panel. Scale bar, 50 μm. (**K**) Immunofluorescence staining and quantification of lipid droplet in transfected epithelial cells following lactate induction. Scale bar, 50 μm. *P < 0.05, **P <0.01, ***P < 0.001, ****P <0.0001.

Given the potential regulation of lactate metabolism by CCT6A, we assessed the functional role of CCT6A in alveolar epithelial cells. We performed extracellular acidification rate (ECAR) analysis as a measure of lactate production by transfecting A549 cells with the CCT6A overexpression plasmid, and the cell real-time energy metabolism analysis data demonstrated that CCT6A led to a sustained decline in ECAR in A549 cells (Figure 2A and B; Supplementary Figure S1). Furthermore, we detected the extracellular lactate level of A549 cells cultured *in vitro* under CCT6A overexpression conditions. The extracellular lactate level was significantly reduced by CCT6A as expected (Figure 2C). Consistently, the extracellular lactate was significantly elevated when CCT6A expression was silenced (Figure 2D). It has been shown that lactate can induce myofibroblast differentiation *in vitro* (Kottmann et al., 2012). Next, we sought to understand whether lactate from epithelial cells stimulates the activation of interstitial cells in a paracrine manner in the lung. We used a coculture system in which fibroblasts and transfected epithelial cells were cultured together (Figure 2E). Fibroblasts cocultured with epithelial cells with CCT6A silencing exhibited observably increased expression of mesenchymal markers (Figure 2F). Immunostaining of α-SMA also showed that the lactate produced in epithelial cells due to the knockdown of CCT6A activated fibroblasts. Monocarboxylate transporter 1 (MCT1) is an important lactate transporter demonstrated to mediate proton-linked bi-directional transport of lactate across the plasma membrane (Hong et al., 2016). Once we treated A549 cells with ARC155858 (a MCT1 inhibitor), it inhibited the efflux of lactate and significantly inhibited the activation of co-cultured fibroblasts (Figure 2G). Collectively, abnormally metabolized epithelial-derived lactate is sufficient to induce the activation of fibroblasts and CCT6A can inhibit the abnormal increase in lactate levels *in vitro.*

According to previous reports, hydroxycarboxylic acid receptor 1 (HCAR1, also known as GPR81) acts as a receptor for lactate, lactate can activate the transcription of HCAR1, and inhibit lipolysis in the adipose tissue (Liu et al., 2009; Ahmed et al., 2010). We asked whether this effect of lactate was present in lung fibrosis. First, cultured epithelial cells were treated with lactate (Sigma, 71718) to detect changes in intracellular lipids. As shown in Figure 2I, the staining of lipid droplets was almost undetectable in the control cells, while the production of lipid droplets in cells treated with lactate was significantly increased. In addition, we detected changes in the level of HCAR1 following CCT6A overexpression, and we observed that CCT6A significantly reduced the transcript levels of HCAR1 (Figure 2H). Next, the impact of CCT6A on lipid metabolism was investigated. Strikingly, higher CCT6A conspicuously reduced intracellular lipid accumulation in A549 cells (Figure 2J and K). These data suggest that CCT6A may reduce the accumulation of intracellular lipids by inhibiting lactate levels.

### Activation of lactate signaling exacerbates the progression of lung fibrosis in mice

**Figure 3:**
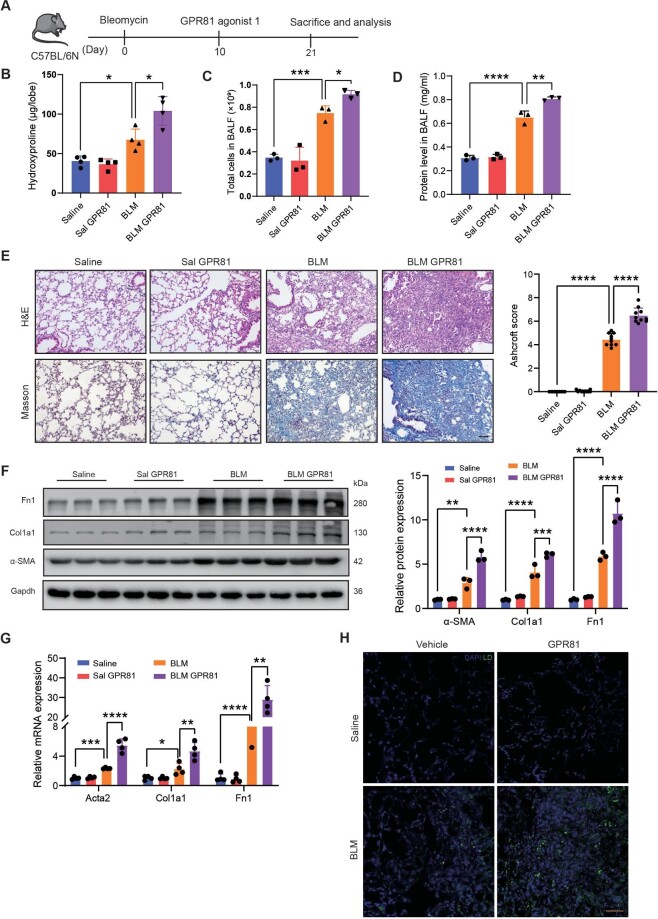
Activation of lactate signal promotes pulmonary fibrosis in mice. (**A**) Timeline of GPR81 agonist-treated mouse lung fibrosis model. (**B**) Quantification of hydroxyproline content in mouse right inferior lobe (*n* = 4 mice per group). (**C**) Quantification of total cells in BALF (*n* = 3). (**D**) Protein concentration in BALF (*n* = 3). (**E**) Representative images of H&E and Masson's Trichrome staining in mouse lung sections (*n* = 3). Scale bar, 50 μm. The Ashcroft score was determined to indicate the severity of fibrosis. (**F**) Western blotting and quantification of fibrosis marker expression in whole lung lysates (*n* = 3). (**G** and **H**) qPCR analysis of α-SMA, Col1a1, and Fn1 mRNA expression levels and immunofluorescence staining of lipid droplet in mouse lung tissue samples (*n* = 4). Scale bar, 50 μm. *P < 0.05, **P <0.01, ***P < 0.001, ****P <0.0001.

To further confirm the regulatory role of CCT6A in lactate and lipid metabolism, as well as to validate the regulation of lactate in lung fibrosis, additional investigations were conducted. We injected a GPR81 agonist (Abmole, M10562) intraperitoneally after mice were treated with BLM to simulate the activation of HCAR1 signaling by lactate *in vivo* (Figure 3A). A significant increase in lung injury and fibrosis was observed in mice following HCAR1 activation, as shown by the hydroxyproline, total cell counts and protein levels of BALF, H&E, and Masson's trichrome staining (Figure 3B–E). To evaluate whether lactate–HCAR1 signaling exacerbates the levels of fibrosis markers in BLM-treated mice, we conducted an assessment of fibrosis gene expression levels using western blotting for protein analysis and quantitative real-time polymerase chain reaction (qPCR) for mRNA analysis. As shown (Figure 3F and G), the expression of pulmonary fibrosis markers was significantly upregulated after BLM treatment, and the activation of HCAR1 exacerbated this change, suggesting that the activation of lactate signaling exacerbates the progression of pulmonary fibrosis *in vivo*. Next, we examined the lipid levels in lung tissue. Lipid droplet staining showed that the HCAR1 agonist significantly increased lipid accumulation in the lung (Figure 3H). These findings indicate that lactate could potentially exert a significant regulation in pulmonary fibrosis through the HCAR1-mediated anti-lipolytic effect.

### CCT6A mediates glycolytic reprogramming by regulating HIF-1α degradation

**Figure 4:**
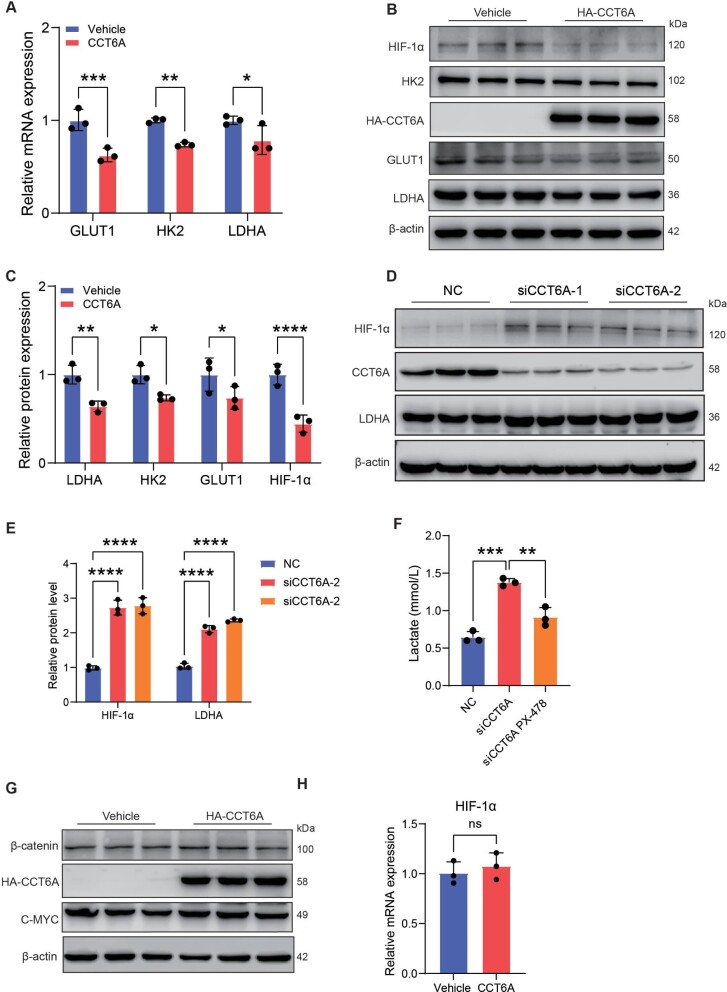
CCT6A mediates a reduction in lactate levels by inhibiting HIF-1α-mediated glycolysis. (**A**) qPCR analysis of mRNA expression levels of glycolysis-related genes GLUT1, HK2, and LDHA in epithelial cells. (**B**–**E**) Western blotting and quantification of glycolysis-related protein expression in A549 cells. (**F**) Extracellular lactate production in siCCT6A-transfected epithelial cells in the presence or absence of PX-478 (15 μM). (**G** and **H**) Western blotting of β-catenin (*P *> 0.05) and C-MYC (*P *> 0.05) protein expression and qPCR analysis of HIF-1α mRNA expression levels (*P *> 0.05) in epithelial cells. *P < 0.05, **P <0.01, ***P < 0.001, ****P <0.0001. ns, not significant.

Lactate is an inevitable product of glycolysis, and glucose undergoes multiple oxidation reactions and finally catalyzes pyruvate to lactate, which is mediated by LDHA. To understand the inhibition of glycolysis by CCT6A, we first analyzed the expression of LDHA mRNA by qPCR, and CCT6A inhibited the expression of the LDHA gene expression (Figure 4A). Additionally, we evaluated the levels of glycolysis-related gene expression. The overexpression of CCT6A significantly reduced the expression of the main glycolysis-related genes in A549 cells (Figure 4A). Consistent results were also obtained from western blot analysis (Figure 4B and C). Notably, the transcription of these genes is regulated by HIF-1α, which is a well-known transcription factor driving glycolysis. Similarly, significantly lower protein levels of HIF-1α were detected in CCT6A-overexpressing cells (Figure 4B and C). Furthermore, we observed a significant upregulation in the protein levels of HIF-1α and LDHA when CCT6A was knockdown (Figure 4D and E), which is consistent with the elevated lactate levels we detected (Figure 2D). To further validate that CCT6A blocks lactate production by inhibiting HIF-1α-mediated glycolysis, we treated cells with PX-478 (a HIF-1α inhibitor) while silencing CCT6A and observed the pharmacological inhibition of HIF-1α significantly attenuated the increase in extracellular lactate levels induced by CCT6A silencing (Figure 4F).

β-catenin and C-MYC have been reported to regulate HIF1-1α and thereby mediate glycolysis reprogramming in disease (Fang et al., 2019). We sought to understand whether the CCT6A-mediated reduction in lactate levels occurs through the β-catenin/cMYC/HIF-1α axis. Nevertheless, overexpression of CCT6A hardly affects β-catenin and cMYC levels (Figure 4G). Indeed, the mRNA levels of HIF-1α were not affected by CCT6A overexpression (Figure 4H). It is well known that the ubiquitin‒proteasome pathway mediates the decomposition of HIF-1α. The protein but not mRNA levels of HIF-1α were much lower in CCT6A-overexpressing cells. These results indicate that that CCT6A may restrain the production of lactate by regulating the steady-state amount of HIF-1α.

### CCT6A interacts with VHL and stimulates the ubiquitination of HIF-1α

**Figure 5:**
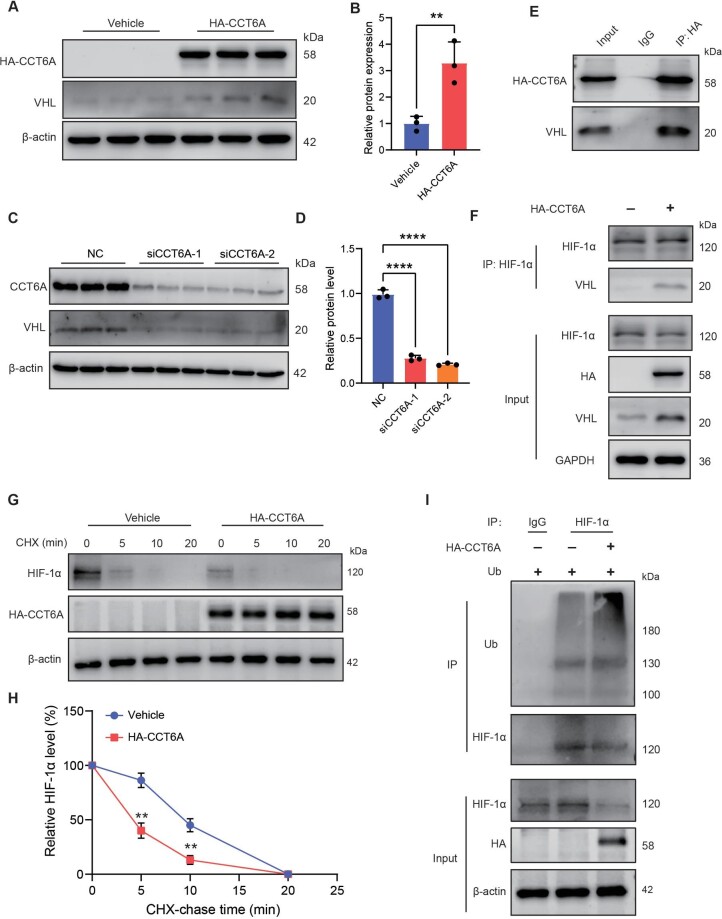
CCT6A interacts with VHL and stimulates the ubiquitination of HIF-1α. (**A** and **B**) Western blotting and quantification of VHL in epithelial cells transfected with vehicle and HA-CCT6A. (**C** and **D**) Western blotting and quantification of VHL in A549 cells transfected with siNC and siCCT6A. (**E**) Co-IP of CCT6A and VHL in epithelial cells. (**F**) Co-IP of HIF-1α and VHL in transfected epithelial cells. (**G** and **H**) A549 cells transfected with HA-CCT6A or vehicle were treated with CHX and collected at the indicated time points for western blotting and quantification of HIF-1α protein expression. (**I**) HEK293T cells were transfected with the indicated plasmids and assayed for the ubiquitination level of HIF-1α by IP and western blotting. **P <0.01, ****P <0.0001.

Ubiquitin-dependent degradation of HIF-1α has been identified previously, and this biological process is mediated by a critical protein known as VHL (Ivan et al., 2001). Moreover, there has been evidence that the chaperone protein TRiC/CCT complex is essential for the stability of VHL (Chesnel et al., 2020). We next investigated whether VHL is regulated by CCT6A by western blotting. As illustrate in Figure 5A and B, CCT6A markedly enhanced the protein levels of VHL, while the knockdown of CCT6A had the opposite effect (Figure 5C and D). Considering that CCT6A, as a chaperone protein, can bind to multiple target proteins to stabilize its conformation, there may be an interaction between CCT6A and VHL quality inspection on the protein‒protein interaction website BioGRID (https://thebiogrid.org/). We then conducted an immunoprecipitation (IP) assay to assess the potential connection between CCT6A and VHL. Notably, there is an evident binding between CCT6A and VHL in A549 cells (Figure 5E), indicating that the interaction between the two could positively regulate VHL. Compelling evidence indicates that VHL recognition and binding to HIF-1α further induce ubiquitination-mediated degradation of HIF-1α. To further verify that CCT6A stimulates the degradation of HIF-1α through VHL. We detected the binding between VHL and HIF-1α after transfection of CCT6A-overexpressing plasmid in A549 cells. IP analysis shown that there was barely binding between VHL and HIF-1α in the control group. In contrast, a prominent combination between VHL and HIF-1α was observed in the CCT6A overexpression group (Figure 5F). To further verify that CCT6A increases the ubiquitination and degradation of HIF-1α, we conducted protein half-life assay. The western blot analysis performed following cycloheximide block of de novo protein synthesis revealed that CCT6A overexpression significantly shortened the endogenous HIF-1α protein half-life in the A549 cells (Figure 5G and H). Furthermore, the ubiquitination assay indicated that CCT6A overexpression increased endogenous HIF-1α ubiquitylation levels relative to the Vehicle group (Figure 5I). Collectively, these results demonstrate that CCT6A interacts with VHL and positively regulates VHL, thus promoting VHL-mediated ubiquitination and degradation of HIF-1α.

### CCT6A attenuates the production of lactate and reverses BLM-induced lung injury and fibrosis in mice

**Figure 6:**
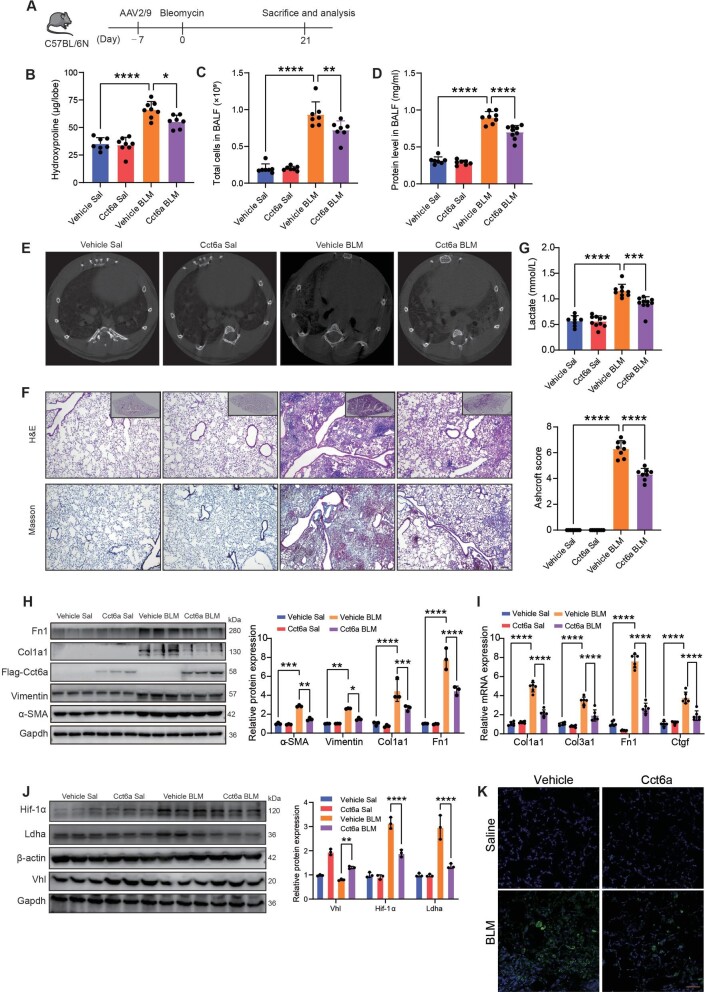
Cct6a restores BLM-induced lung fibrosis in mice. (**A**) Timeline of AAV-treated mouse lung fibrosis model. (**B**) Hydroxyproline in the right inferior lobe (Vehicle Sal *n* = 7, Cct6a Sal *n* = 8, Vehicle BLM *n* = 8, Cct6a BLM *n* = 7). (**C**) Total cell counts in BALF (*n* = 7 mice per group). (**D**) Protein concentration in BALF (Vehicle Sal *n* = 7, Cct6a Sal *n* = 7, Vehicle BLM *n* = 8, Cct6a BLM *n* = 9). (**E**) Representative images of micro CT scans of mouse lung density (*n* = 3). (**F**) Representative images of H&E and Masson's Trichrome staining in mouse lung sections (*n* = 3). Boxed regions in the right superior panel are panoramic images of the lung tissue sections. Scale bar, 50 μm. The Ashcroft score was determined to indicate the severity of fibrosis. (**G**) Lactate level in mouse serum (Vehicle Sal *n* = 8, Cct6a Sal *n* = 10, Vehicle BLM *n* = 9, Cct6a BLM *n* = 10). (**H**) Western blotting and quantification of fibrosis marker expression in whole lung lysates (*n* = 3). (**I**) qPCR analysis of Col1a1, Col3a1, Fn1, and Ctgf mRNA expression levels in the lung homogenate (*n* = 6). (**J**) Immunoblot analysis of Vhl, Hif-1α, and Ldha protein levels in whole lung lysates (*n* = 3). (**K**) Immunofluorescence staining of lipid droplet in mouse lung sections (*n* = 3). Scale bar, 50 μm. *P < 0.05, **P <0.01, ***P < 0.001, ****P <0.0001.

To explore the regulation of CCT6A in the pathogenesis of lung fibrosis *in vivo*, we subjected adeno-associated virus serotype 2/9 (AAV2/9), either pcAAV-CMV-Cct6a-Flag or pcAAV-CMV to C57BL/6N mice via intratracheal delivery, and then intratracheal BLM or saline to the mice a week later (Figure 6A). Microcomputed tomography scans for mice were performed before sacrifice and showed a significant increase in hyperdense areas in the lungs of BLM-injected mice compared with controls. However, in the lungs of Cct6a-overexpressing mice, the presence of hyperdense areas remained extensive, but their volume and density decreased (Figure 6E). Notably, compared with the saline group, BLM remarkably induced lung parenchymal fibrotic lesions in mice, while the administration of AAV2/9-Flag-Cct6a attenuated BLM-mediated lung fibrosis, as shown by hydroxyproline assays, H&E and Masson's trichrome staining (Figure 6B and F). Instead, knockdown of Cct6a in mice significantly exacerbated BLM-induced lung injury (Supplementary Figure S2A and C). The exacerbated fibrosis due to Cct6a knockdown was further supported by increased fibronectin in the lung, as analyzed by immunohistochemistry (Supplementary Figure S2C). Consistently, overexpression of Cct6a resulted in a reduction in total leukocytes and total protein concentration collected from BALF (Figure 6C and D). We next asked whether Cct6a-mediated fibrosis attenuation regulated the production of lactate in mice. As previously observed, the level of lactate in BLM-treated mice was markedly increased in mouse serum, and Cct6a reversed this phenomenon (Figure 6G). Knockdown of Cct6a increased lactate levels in BLM-induced mouse serum (Supplementary Figure S2E). These results suggest that Cct6a inhibits the progression of pulmonary fibrosis in mice by reducing the increase of lactate level induced by BLM.

To further verify the inhibitory influence of overexpression of Cct6a on lung fibrosis in the mice injected BLM, we detected the expression levels of lung fibrosis related genes. As illustrated in Figure 6H and I, compared with control mice, overexpression of Cct6a significantly inhibited the elevation of Fn1, Ctgf, Col1a1, and α-SMA induced by BLM, either mRNA or protein levels. However, knockdown of Cct6a in mice resulted in a collective increase in fibrosis markers in the lung (Supplementary Figure S2B and D). Additionally, BLM significantly induced elevated of HIF-1α levels in the lung, while overexpression of Cct6a reduced the protein level of HIF-1α in mice (Figure 6J). Consistently, high levels of VHL were noted in the Cct6a-overexpressing mice (Figure 6J). Furthermore, we measured the lipid level in the lungs of mice. Staining for lipid droplets in lung sections showed BLM treatment significantly increased the accumulation of lipid in the lungs but decreased lipid levels in Cct6a-overexpressing mice (Figure 6K). Prospectively, the loss of Cct6a increased BLM-induced lipid accumulation in the lungs (Supplementary Figure S2F). Collectively, our results imply that Cct6a weaken the levels of lactate and lipid in the fibrotic lung and protects mice from BLM-induced lung injury and fibrosis.

## Discussion

**Figure 7:**
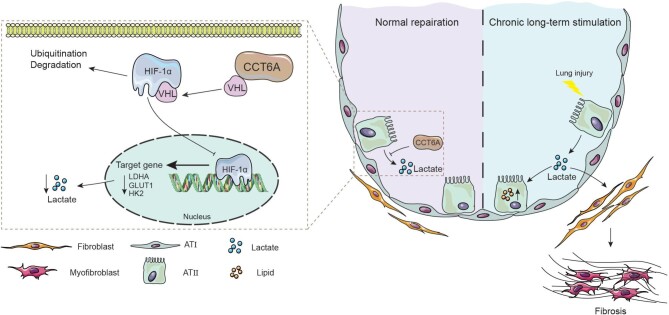
Mechanism of CCT6A preventing the progression of pulmonary fibrosis. In the fibrotic lung, metabolic abnormalities in alveolar epithelial cells lead to an increase in HIF-1α-mediated lactate production, which induces the activation of interstitial fibroblasts, thereby accelerating the progression of pulmonary fibrosis. High expression of CCT6A stabilizes the cellular VHL protein level and thus promotes VHL-mediated ubiquitination and degradation of HIF-1α, thereby inhibiting the increased lactate level in alveolar epithelial cells and alleviating pulmonary fibrosis.

Despite recent advancements in understanding IPF and the identification of potential therapeutic drugs to mitigate lung function decline, there is still a significant lack of clear and effective treatments for this condition. Therefore, there is an urgent need for an enhanced understanding of the underlying pathogenesis of IPF. Our results show that lactate increases lipid accumulation in AEC2s, and the activating lactate signaling exacerbates BLM-induced lung injury and fibrosis in mice. In addition, we illustrated a crucial regulation for CCT6A in lactate signaling and the progression of lung fibrosis. CCT6A is increased in the fibrotic lungs compared with normal, and CCT6A levels were significantly correlated with disease severity. We found that abnormally metabolized epithelial-derived lactate is sufficient to induce the activation of fibroblasts. Intervention of CCT6A significantly inhibits the production of lactate and lipids in AEC2s. Mechanistic experiments revealed that CCT6A could combine with VHL, after which VHL bound specifically to HIF-1α and stimulated its ubiquitination and degradation, by which it attenuated the generation of lactate and blocked the lactate-HCAR1 signaling to promote the lipolysis of AEC2s (Figure 7). Following the observation that CCT6A could regulate lactate metabolism in cells cultured *in vitro*, we next investigated the regulation of CCT6A in experimental murine lung fibrosis. Overexpression of the adeno-associated virus CCT6A protects mice against lung injury, fibrosis and the production of lactate induced by BLM. Taken together, we further discovered the regulatory mechanism of lactate in pulmonary fibrosis and demonstrated that targeting CCT6A may be a viable strategy for treating pulmonary fibrosis.

While abnormal cell metabolism is known to be involved in various pathological processes, there have been limited efforts to identify metabolic disorders specifically related to organ fibrosis, such as IPF. The metabolic processes occurring in the fibrotic lung have not been thoroughly characterized, and there is a lack of therapeutic approaches based on metabolic interventions for treating fibrotic diseases. Notably, growing evidence suggests a connection between abnormal lactate metabolism and the development of several diseases. Compelling evidence suggests that lung lactate levels in IPF patients are significantly elevated. Lactate may play a role in stimulating fibroblast differentiation and regulating epigenetics (Kottmann et al., 2012; Cui et al., 2021). Previously, lactate was primarily believed to have functions limited to the interstitium. However, there is a need for a better understanding of the factors that regulate lactate metabolism in the lung. The disruption of mitochondrial energy metabolism in IPF-derived AEC2s results in an imbalance in intracellular lactate metabolism, which could be a key contributor to the increased lactate levels in the lung (Newton et al., 2021). Moreover, as a signal molecule called ‘lactomone’, whether the lactate produced by AEC2s regulates the fate of lung cells through autocrine or paracrine signaling should also be our focus. Lactate can mediate the inhibition of G protein signaling through its receptor HCAR1, thereby inhibiting the decomposition of intracellular lipids in adipose tissue (Liu et al., 2009; Ahmed et al., 2010). Interestingly, one of the characteristics of lung fibrosis is the accumulation of lipids in alveoli. Although researchers have suggested that lactate may drive more diverse events in the lung niche (Tuder et al., 2012), and lactate is reported as a major source of lipids (Chen et al., 2016; Faubert et al., 2017; Pucino et al., 2019). However, it is still unknown whether there is an inevitable relationship between the increase in lactate and lipids in fibrotic lungs. We found an increased lipid droplets in A549 cells following lactate stimulation, and the activation of lactate-HCAR1 signaling aggravated BLM-induced lung fibrosis *in vivo*, while significantly enhancing the accumulation of lipids in lungs. The results indicate a potential association between the elevated levels of lactate and lipids in fibrotic lungs.

CCT6A is a member of the chaperonin containing TCP1 complex (CCT), also known as the TCP1 ring complex (TRiC). The TCP1 complex family members have been reported to be involved in the folding of approximately 20% of the target protein in the organism, involving various biological processes within cells (Freund et al., 2014). Especially CCT6A is upregulated in many pathological conditions. In fact, CCT6A participates in cell cycle progression and cytoskeletal organization (Brackley and Grantham 2009; Zeng et al., 2019) and in multiple signaling pathways involved in cell proliferation (Xiang et al., 2008; Zeng et al., 2022). CCT6A is considered a key regulator of several cancers and a newly discovered clinical biomarker which is associated with survival and prognosis of patients (Huang et al., 2019; Ma et al., 2021; Wang et al., 2022; Yang et al., 2022). As a SMAD2-specific interaction protein, CCT6A acts as an inhibitor of SMAD2 in NSCLC, thereby promoting TGF-β1-mediated NSCLC transfer (Ying et al., 2017). Although CCT6A as a major regulation factor in tumor development, the detailed regulatory mechanism is still lacking. Our previous research suggests a potential association between CCT6A and elevated lactate levels in IPF patients, but its exact regulation in IPF is still unclear (Wang et al., 2023).

Herein, our results show aberrantly increased CCT6A expression in AEC2s from IPF patient lung sections. Similarly, CCT6A highly expressed in AEC2s derived from mice treated BLM. We next focused on assessing the regulation of lactate metabolism by CCT6A in AEC2s. Interestingly, the overexpression of CCT6A remarkably led to a decrease in the ECAR as a measure of lactate production in A549 cells. In addition to altered lactate metabolism, CCT6A inhibited the accumulation of intracellular lipid droplets. Mesenchymal-epithelial crosstalk is critical to the process of lung fibrosis. We attempted to investigate whether lactate derived from epithelial cells can promote the activation of fibroblasts *in vitro*. Therefore, we constructed an epithelial-mesenchymal cell co-culture system, and fibroblasts cultured with CCT6A-silenced epithelial cells exhibited obvious fibroblast-myofibroblast transformation. Furthermore, we studied how CCT6A expression inhibited the generation of lactate *in vitro*. The overexpression of CCT6A robustly decreased the levels of glycolysis-related genes (HK2, GLUT1, and LDHA). Notably, CCT6A inhibited the protein level of HIF-1α, and the silencing of CCT6A leads to up-regulation of HIF-1α protein levels, which is an important transcription factor regulating glycolysis. Furthermore, pharmacological inhibition of HIF-1α reversed the increase in extracellular lactate levels caused by CCT6A knockdown, further indicating that CCT6A may regulate the glycolysis of AEC2s through HIF-1α.

One of many key gene changes in disease is the elevated level HIF-1α activity, accompanied by the loss of VHL (Semenza, 2013). It has been reported that VHL mutations cause pulmonary hypertension and pulmonary fibrosis in aged mice (Hickey et al., 2010). Other study shown that highly expressed VHL promoted the proliferation of fibroblasts and increased the deposition of ECM (Zhou et al., 2011). Proverbially, VHL can recognize and combine with HIF-1α and subsequently degrade HIF-1α by the ubiquitin-dependent proteasome pathway (Maxwell et al., 1999). Given the regulation of VHL on HIF-1α, and CCT6A did not regulate HIF-1α at the transcriptional level. We speculated that CCT6A reduced the protein levels of HIF-1α through VHL and then decreased lactate levels in A549 cells.

It has been reported that eukaryotic chaperonin TRiC/CCT mediates folding of VHL (Feldman et al., 2003). Therefore, we examined the regulatory effect of CCT6A on VHL. Strikingly, CCT6A interacts with VHL and positively regulates the protein level of VHL. Knockdown of CCT6A leads to a decrease in VHL protein levels. And our further results indicated that the overexpression of CCT6A enhanced the binding of HIF-1α and VHL, indicated increased protein degradation of HIF-1α. The protein half-life experiment also showed that CCT6A accelerated the degradation of endogenous HIF-1α. Moreover, we found that CCT6A reduced the stability of HIF-1α proteins by increasing its polyubiquitination. Finally, we evaluated the efficacy of targeted Cct6a in mice with BLM-induced fibrosis. As a result, mice overexpressing Cct6a showed significant attenuation of BLM-induced pulmonary fibrosis, including decreased lactate levels in serum and decreased lipid accumulation in the lungs, further demonstrating the therapeutic potential of CCT6A as a possible clinical target.

Our study has several limitations. Firstly, although our data showed that CCT6A bound to VHL, there is still no conclusive evidence that CCT6A can stabilize VHL independently or through the TCP1 complex. Secondly, about 10% of proteins fold correctly through TCP complex in eukaryotic organisms. Therefore, the inhibition of glycolysis by CCT6A may involve other mechanisms independent of HIF-1α in AEC2s. However, from another point of view, our results are sufficient to prove the inhibition of CCT6A on glycolysis, which is a potential target for the treatment of pulmonary fibrosis. Finally, in this study, we focused on the role of CCT6A in epithelial cells. While the results of CCT6A in mice show positive outcomes, it is important to consider that ACE2s play a central role in the pathogenesis of pulmonary fibrosis. Additionally, CCT6A may display inconsistent phenotypes in both epithelial cells and fibroblasts. Therefore, further studies are necessary to fully understand the underlying mechanism.

Collectively, we first elucidated the potential connection between lactate and lipids in the context of pulmonary fibrosis. Furthermore, the current study discovered that a significant upregulation of CCT6A in fibrotic lungs, and associated with lactate metabolism in AEC2 cell. *In vitro* researches proved CCT6A positively regulates VHL and promotes the binding between HIF-1α and VHL, thus promoting the degradation of HIF-1α and suppressing lactate and lipid levels. More importantly, overexpression of CCT6A in mice significantly inhibits BLM-induced lung fibrosis. In summary, this research contributes to a better understanding of pulmonary fibrosis pathogenesis and identifies a promising avenue for further investigation and treatment that the gene targeted to regulate lactate metabolism may be an effective treatment for pulmonary fibrosis.

## Materials and methods

### Clinical samples

IPF lung tissue samples were obtained from the remains of a patient's surgical biopsy or lung transplant. The control groups were taken from normal lesion-free edge tissue of the lung cancer specimen. IPF was diagnosed according to consensus criteria based on ATS/ERS/JRS/ALAT Clinical Practice Guidelines (Raghu et al., 2018). Clinical information for all patients was described previously (Wang et al., 2022). All studies were approved by Henan Provincial Chest Hospital Medical Research Ethics Committee (No. 2020-03-06). The research conformed to the principles of the Declaration of Helsinki. Oral and written informed consent was obtained from all patients.

### Animals

Sftpc^CreERT2^ mice and ROSA26^tdTomato^ mice were purchased from Cyagen Biosciences. Background C57BL/6N was from Charles River. All mice were cared in pathogen-free lab and provided normal living conditions. Animal husbandry and experimental procedures were performed following the guidelines of the Animal Care and Use Committee of Henan Normal University (IACUC, SMKX-2118BS1018), which in accordance with the guidelines of Animal Behavior Associations and national regulations.

### BLM-mediated induction of pulmonary fibrosis

Male mice of 8 weeks of age were used for subsequent experiments. Anesthetized mice were randomly assigned to BLM (1.5 U/kg) or saline alone via orotracheal instillation. On Day 21 after administration, lungs were collected for subsequent assays. The trachea was cannulated and washed once with 0.8 ml sterile PBS, and the lavage solution were directly used for cell counting, and the cell-free supernatants were used for protein concentration detection.

### Hydroxyproline assay

Quantification of collagen in the lung by hydroxyproline assay kit from Sigma (MAK008). Hydroxyproline in the lungs was quantified following manufacturer's instruction. Data are showed as μg of hydroxyproline/lobe.

### Pathological staining

H&E and Masson staining were performed on the lung slices following the manufacturer's protocols. Panoramic images of the stained sections were acquired with the Cytation 10-confocal imaging reader (BioTek). The modified Ashcroft method was used to score the degree of lung fibrosis in mice. In general, the severity of fibrosis in lung tissue sections was evaluated according to average of the severity scores of the observed microscopic visual field. The 10 randomly selected areas in every mouse lung were scored, and the average scores were displayed in an image magnified by 100-fold magnification. Three independent observers were scored by blind method.

### Immunohistochemistry and immunofluorescence

Lung tissue sections were paraffin removal and blocking, immune staining for CCT6A and FN1 was performed. Then, the slices were incubated with primary antibody and secondary antibodies, respectively. Finally, the sections were incubated with DAB reagent and viewed with light microscopy. For the Immunofluorescence staining for frozen sections, after fixation of lung tissue in 4% paraformaldehyde, transferred to 15% and 30% sucrose for 12 h, respectively, and then embedded in OCT. Frozen sections were incubated with primary antibody and secondary antibodies (1:1000, Cell Signaling Technology, 4408, 4412, 8890, 8889, respectively). DAPI (Solarbio) was used to counterstain the nuclei and then observed by confocal laser scanning microscopy (Leica).

### Cell culture

The human AT2-like cell line (A549) and human fibroblast cell line (MRC-5) were purchased from the ATCC (CCL-185 and CCL-171, respectively). Cells were cultured in MEM and DME/F-12 (Hylone) supplemented with 10% FBS and a 1% penicillin and streptomycin at 37°C in 5% CO_2_.

### Overexpressing and silence of CCT6A

HA-tagged CCT6A were cloned into pcDNA3.1 vector by a standard subcloning procedure. The CCT6A siRNA and a negative control were synthesized by RiboBio. The siRNA specific for CCT6A targeted the following sequence: si#1: 5′-GTGTCATTAGAGTATGAGA-3′ or si#2: 5′-CATACATCCTCACTTGTAA-3′. Cells were transfected with the plasmid or siRNA following to the manufacturer's instruction. After 24 or 48 h, the transfected cells were used for follow-up experiments.

### Glycolysis stress test

In order to evaluate the change in the ECAR, glycolytic rate and glycolytic capacity were assessed using Seahorse XF96 Extracellular Flux Analyzer (Seahorse Bioscience). Briefly, 20000 cells/well were seeded into the XF96 cell culture microplate and transfected. After the assay began, glucose, oligomycin, and 2-DG were added in sequence.

### qPCR

TRlzol reagent (TaKaRa) was used for extracting total RNA and the GoScript™ Reverse Transcription System (A5001, Promega) was used for the synthesis of complementary DNA. The synthesized cDNA was used for subsequent qPCR in the LightCycler 480 Real-Time PCR system (Roche). Each reaction was performed in triplicate. The RNA levels of β-actin were employed as an internal standard control. All primer sequences were provided in Table 1.

**Table 1:** Primers and oligonucleotides.


HA-tagged CCT6A	
HA-CCT6A	F: CGCGGATCCATGGCGGCGGTGAAGACCCTGAAC
	R: CCGCTCGAGTCAAGCGTAGTCTGGGACGTCGTATGGGTAACCTTTCAGAGAAGACAT
HA-Cct6a	F: CGCGGATCCATGGCGGCGGTAAAGACCCTAAAT
	R: CCGCTCGAGTCAAGCGTAGTCTGGGACGTCGTATGGGTAACCCTTCAGAGAGGACA
Primers used for qPCR (human)	
CCT6A	F: TGCATTGCTCATTATTCCCA
	R: CACAATAGTTATCCCATACGCC
HCAR1	F: GCCTGCCTTTTCGGACAGACTA
	R: ACCACCGTAAGGAACACGATGC
HIF-1α	F: TATGAGCCAGAAGAACTTTTAGGC
	R: CACCTCTTTTGGCAAGCATCCTG
LDHA	F: GGCCTGTGCCATCAGTATCT
	R: AGATATCCACTTTGCCAGAGACA
GLUT1	F: TTGCAGGCTTCTCCAACTGGAC
	R: CAGAACCAGGAGCACAGTGAAG
HK2	F: GAGTTTGACCTGGATGTGGTTGC
	R: CCTCCATGTAGCAGGCATTGCT
β-actin	F: AGAAAATCTGGCACCACACC
	R: TAGCACAGCCTGGATAGCAA
Primers used for qPCR (mouse)	
Cct6a	F: GCGAAAGAAGGGATCGTAGCTC
	R: AAGCCCTGCATGTCCCAAACAG
Col1a1	F: CCTCAGGGTATTGCTGGACAAC
	R: CAGAAGGACCTTGTTTGCCAGG
Col3a1	F: GACCAAAAGGTGATGCTGGACAG
	R: CAAGACCTCGTGCTCCAGTTAG
Fn1	F: CCCTATCTCTGATACCGTTGTCC
	R: TGCCGCAACTACTGTGATTCGG
Ctgf	F: TGCGAAGCTGACCTGGAGGAAA
	R: CCGCAGAACTTAGCCCTGTATG
β-actin	F: GTACCCAGGCATTGCTGACA
	R: AACGCAGCTCAGTAACAGTCC

### Immunoblotting

Total proteins were collected from mouse tissues and cultured cells. After separating in SDS–PAGE, the proteins were incubated whit primary antibody and secondary antibodies in sequence, followed by visualization and gray values analysis using the Odyssey Fc Imaging System (LI-COR Bio). The details of the antibodies are listed in Table 2.

**Table 2:** List of antibodies used in this study.

**Designation**	**Species**	**Source**	**Dilution**
CCT6A	Polyclonal rabbit	Affinity, DF4562	1:1000
LDHA	Polyclonal rabbit	Affinity, DF6280	1:1000
VHL	Polyclonal rabbit	Proteintech, 24756-1-AP	1:1000
GLUT1	Polyclonal rabbit	Proteintech, 21829-1-AP	1:1000
HK2	Polyclonal rabbit	Proteintech, 22029-1-AP	1:1000
FN1	Polyclonal rabbit	Proteintech, 15613-1-AP	1:2000
HA-tag	Monoclonal rabbit	Cell Signaling Technology, 2130	1:2000
HIF-1α	Monoclonal rabbit	Cell Signaling Technology, 36169	1:1000
COL1A1	Monoclonal rabbit	Cell Signaling Technology, 72026	1:2000
COL1A1	Polyclonal rabbit	Proteintech, 14695-1-AP	1:2000
α-SMA	Polyclonal rabbit	Abcam, ab5694	1:2000
Ubiquitin	Monoclonal mouse	Cell Signaling Technology, 3936	1:1000
β-ACTIN	Monoclonal mouse	Affinity, T0022	1:100000
GAPDH	Monoclonal mouse	Proteintech, HRP-60004	1:10000
Goat anti-Rabbit IgG		JacksonimmunoResearch, 111-035-003	1:10000
Goat anti-Mouse IgG		JacksonimmunoResearch, 115-035-003	1:10000

### Lactate assay

Quantification of the extracellular and serum lactate using lactate assay kits (MAK064, Sigma). Appropriate amount of culture medium or serum were deproteinized to filtrate lactate dehydrogenase. After adding components in the reagent kit to the sample, measure the absorbance value at 570 nm.

### Lipid staining

Frozen sections and cultured cells were incubated in 60% isopropanol for 2 min. Next, samples were incubated in Oil Red O solution for 5 min, differentiated by 60% isopropanol for 1 min, and then washed with ddH_2_O. Finally, the nuclei were counterstained with hematoxylin, coverslip with 50% glycerol, and observed with light microscopy. For the fluorescence staining of lipid droplets, samples washed with PBS to remove the embedding agent or culture medium, incubate with appropriate concentration of Lipi-Green (LD02, DOJINDO) at 37°C for 30 min. DAPI was used to counterstain the nuclei and then examined by confocal laser scanning microscopy.

### IP assay

As previously described, 48 h after transfection of epithelial cells with HA-CCT6A plasmid, we performed Collecting whole-cell lysate, and incubated with the specific antibodies. Next, all samples were Protein A/G Plus-Agarose (88803, Thermo Fisher Scientific) incubated with ag at room temperature for 4 h. The interaction complexes were separate d from the beads and then analyzed by western blotting.

### Statistical analyses

Data were assessed by using Prism 9.0 software (GraphPad Software). The control and experimental groups should include at least three biological replicates. Error bars present mean ± standard deviation (SD), which was used to describe the variability among biological replicates. Student's *t*-test was used to compare two groups of data, and ANOVA plus Tukey was used for multiple comparison test.

### Data availability

All high-throughput data mentioned in the article are publicly available at Gene Expression Omnibus (GEO) under the accession number GSE47460. All other data that support the findings of this study are available from the corresponding author upon request.

## Supplementary Material

mjae021_Supplemental_File

## Notes

**Author contributions:** P.Y. performed most experiments with the assistance of K.Y., M.Z., Y.D., and X.P. W.L., and M.X. performed animal experiments. L.L. and C.L. performed the histologic analysis. Z.L. and L.W. performed the statistical analysis. P.Y. wrote the manuscript. G.Y. interpreted results and revised the manuscript.
