# Supplementary material for: CCT6A alleviates pulmonary fibrosis by inhibiting HIF-1α-mediated lactate production
Source: J Mol Cell Biol. 2024 May 17;16(5):mjae021. doi: 10.1093/jmcb/mjae021 (PMC11574388; doi:10.1093/jmcb/mjae021)
Supplement: mjae021_Supplemental_File [file mjae021_supplemental_file.pdf]

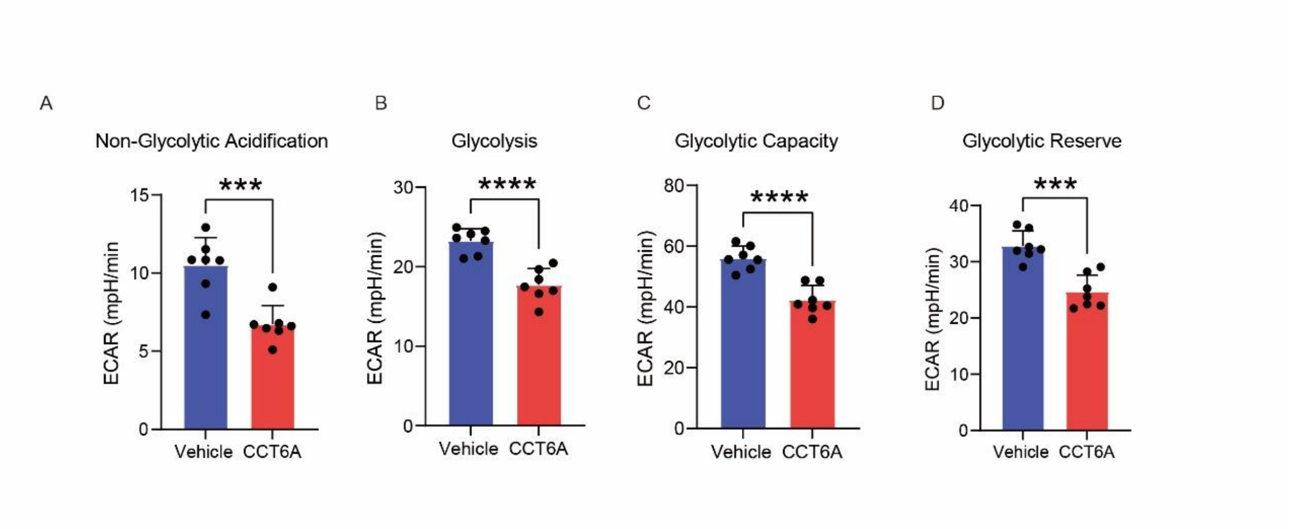

Supplementary Figure S1 Bar charts of glycolysis stress test for AEC2s transfected with CCT6A plasmid. ***P <0.001, ****P < 0.0001.

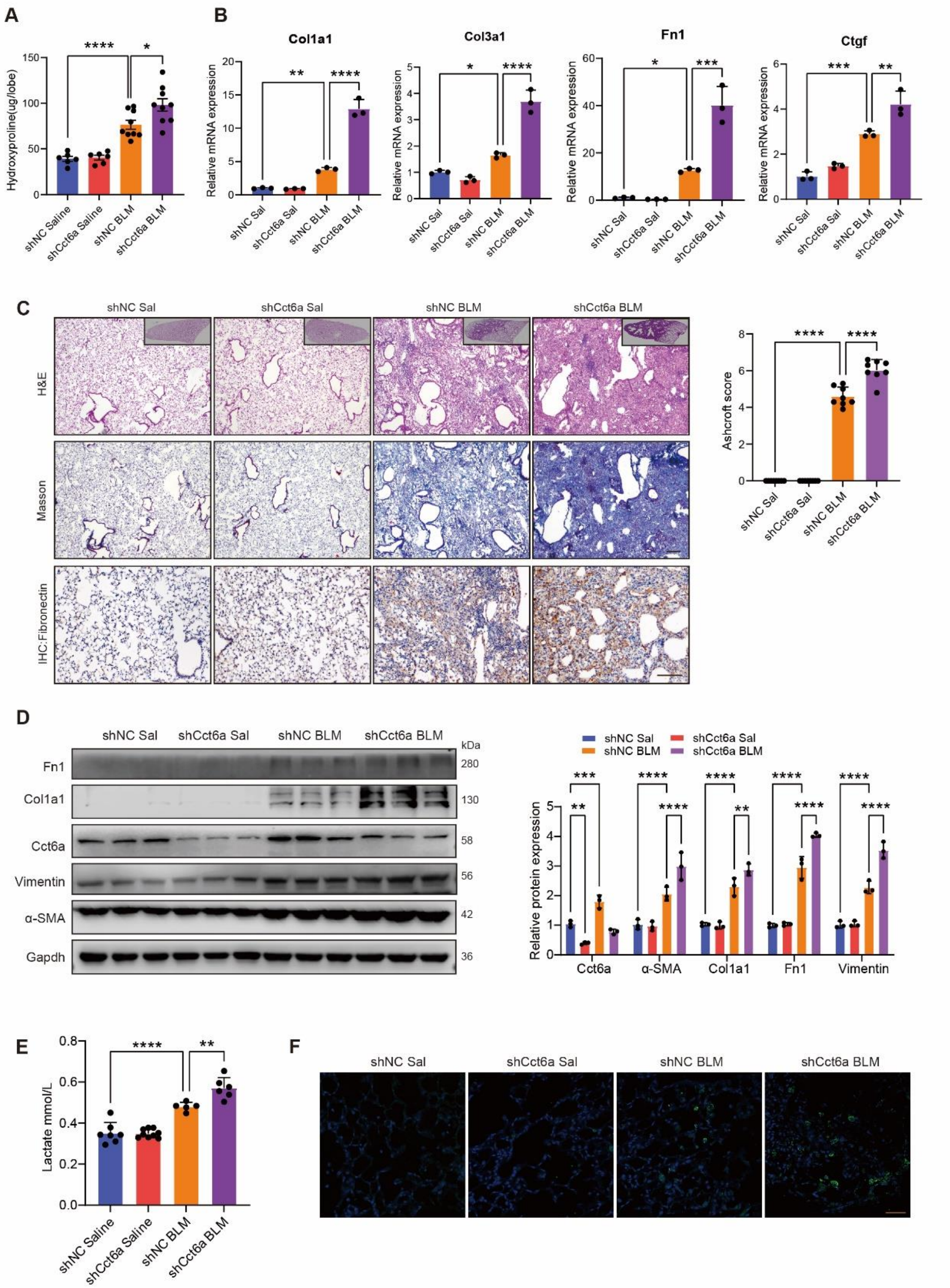

Supplementary Figure S2 The knockdown of Cct6a exacerbates bleomycin-induced pulmonary fibrosis in mice. (**A**) Quantification of hydroxyproline in the right inferior lobe of mice (shNC Sal n

= 6, shCct6a Sal n = 6, shNC BLM n = 9, shCct6a BLM n = 9). (**B**) RT-PCR analysis of Col1a1, Fn1 and Ctgf expression in the lung homogenate. (**C**) H&E and Masson's Trichrome staining of representative lung sections from mice. Boxed regions in the right superior panel are panoramic images of the lung tissue sections. Right panel shows the quantitative mean score of the severity of fibrosis. Immunohistochemical staining of FN1 is located below of the panel. Scale bar, 50 μm. (**D**) Western blot analysis of Fn1, α-SMA, Col1a1, Vimentin, and Cct6a in whole lung lysates (n=3). (**E**) Quantification of lactate level in serum of mice (shNC Sal n = 7, shCct6a Sal n = 9, shNC BLM n = 5 and shCct6a BLM n = 6). Scale bar, 50 μm. (**F**) Immunofluorescence staining of lipid droplet in representative lung sections from mice (n=3). Scale bar, 50 μm. *P<0.05, **P <0.01, ***P<0.001, ****P <0.0001.
